# Symptomatic bilateral Morgagni diaphragmatic hernias in an elderly patient

**DOI:** 10.1259/bjrcr.20160110

**Published:** 2017-03-10

**Authors:** Cathal O'Brien, J Craig Jobling

**Affiliations:** Department of Radiology, Nottingham University Hospitals NHS Trust, Nottingham, UK

## Abstract

We present a rare case of bilateral Morgagni hernias in an elderly female patient. She initially presented with coffee-ground vomiting and underwent an OGD from which she was given a diagnosis of oesophagitis. On her second presentation (a year later), CT was performed after OGD to evaluate what was thought to be a complex hiatus hernia causing haematemesis, at which point a diagnosis of bilateral Morgagni hernias was made. Surgical management with laparoscopic mesh repair was performed and she made an uneventful recovery.

## Clinical presentation

An 83-year-old lady initially presented to hospital a year prior to diagnosis with a 3-week history of dyspepsia and a single episode of coffee-ground vomit. She was haemodynamically stable at that time and physical examination was unremarkable. She was admitted under gastroenterology and received an inpatient OGD, which reached the D2 duodenal segment and demonstrated distal oesophagitis. Subsequently, she was started on omeprazole and recommended for repeat OGD as an outpatient. No imaging was performed at this admission. In absence of any further symptoms she was discharged.

Repeat OGD was performed 2 months later and also reached the D2 duodenal segment, demonstrating good resolution of the oesophagitis and making note of a new hiatus hernia.

A year after the last OGD this lady re-presented to ED with severe, sharp, left-sided abdominal pain, with an episode of coffee-ground vomit. Once in ED she had a further four episodes of coffee-ground vomiting amounting to roughly 750 ml. On physical examination she was haemodynamically stable, with unremarkable chest auscultation and a soft, non-tender abdomen. Blood tests were unremarkable, except for a slightly raised WCC (15.4) which was felt to be in keeping with her history of concurrent coryzal illness. Past medical history comprised of poorly controlled hypertension and nil else of note.

She was again admitted to gastroenterology and OGD repeated. On this occasion the scope could not be passed beyond the stomach due to twisting of the presumed hiatus hernia complicating the anatomy.

## Differential diagnosis

- Gastric volvulus;
- Gastric outlet obstruction;
- Diaphragmatic hernia.

## Investigations/imaging findings

**Figure 1.**
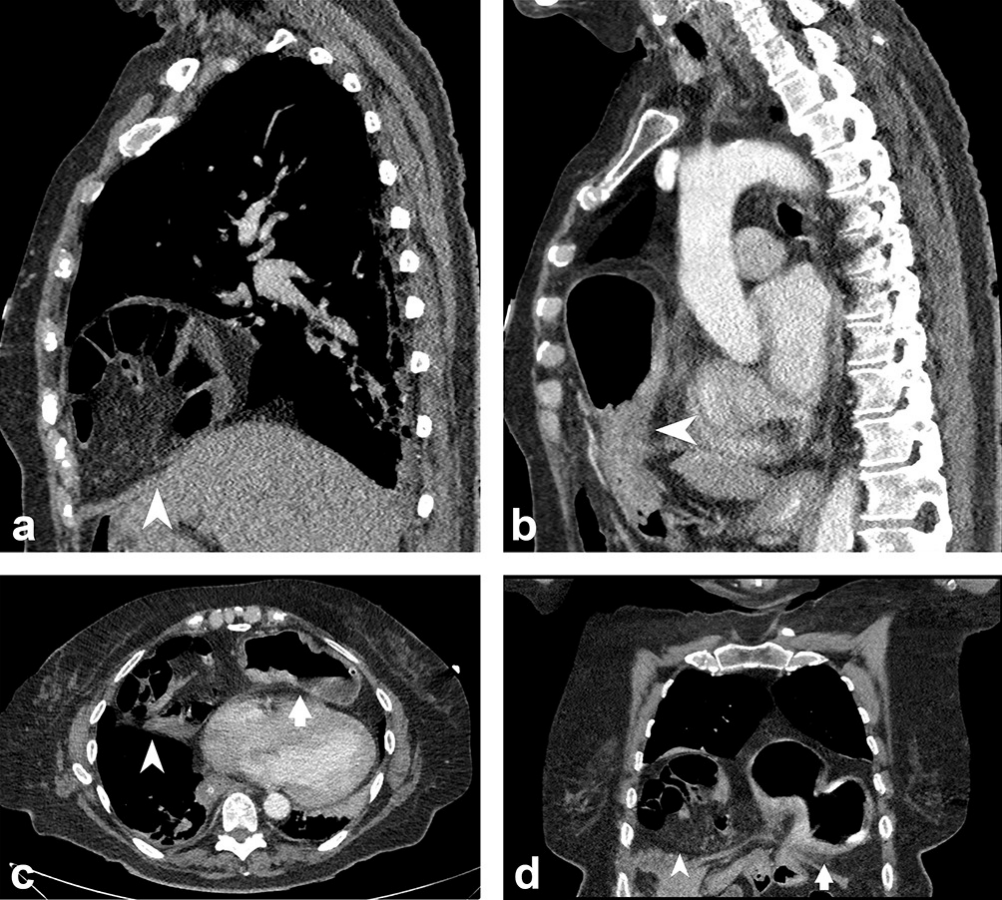
CT images demonstrating (a) herniation of small bowel and mesentery through the right diaphragmatic defect (arrowhead); (b) stomach herniating through the left defect (arrowhead). Both the right (arrowhead) and left (arrow) diaphragmatic defects are demonstrated together in (c) axial and (d) coronal planes.

The patient’s care was passed to the General Surgeons who requested CT imaging. Prior to this the patient had undergone no thoracic or abdominal imaging either with CT or plain radiography. CT demonstrated bilateral anterior diaphragmatic (Morgagni) hernias, with herniation of the stomach into the left defect and herniation of small bowel and mesentery into the right defect (Figures 1a–d).

## Treatment

With gastric decompression via NG tube and NBM her symptoms settled and initially conservative management was planned with reference to her anaesthetic risk. However, after several days she remained unable to tolerate oral intake and bilateral laparoscopic mesh repair was performed. Recovery was uneventful and she was discharged home after several days.

## Discussion

Anterior diaphragmatic hernias were first described in 1769 by Italian anatomist and pathologist Giovanni Battista Morgagni. They are located posterolateral to the sternum and arise through an area of diaphragmatic weakness (Larry space). Most commonly they contain omentum or less frequently transverse colon or stomach.

Congenital diaphragmatic hernias are uncommon and within this group anterior (Morgagni) hernias are the least common type, accounting for only 3% of presentations. Right-sided Morgagni hernias are by far the most common subtype at 90% of cases. Bilateral Morgagni hernias in adults are very rare, with only a handful reported in the last decade.^1–5^

Despite being classified as ‘congenital’, anterior diaphragmatic hernias more commonly present in adulthood, with an increasing number of case reports of unilateral hernias in elderly patients.^1–3^ This is thought to be due to episodes of raised intra-abdominal pressure such as coughing or vomiting eventually causing herniation through the congenital weakness.^4^ As such, obesity, pregnancy and abdominal trauma are risk factors.^5^ In the few prior case reports of bilateral hernia in adults the age of presentation is typically lower (4th to 5th decade); our patient was unique in presenting so late in life, having been completely asymptomatic up until her 9th decade.^6,7^

It was believed that the majority of Morgagni hernias are asymptomatic and detected incidentally.^8^ However a larger case series more recently has shown that the majority of patients (over 70%) do present with symptoms related to their hernia, usually respiratory related from mass effect of herniated abdominal organs into the thoracic cavity. Other presentations include bloating, vomiting and more acute presentations with abdominal pain, GI haemorrhage, obstruction or strangulation.^9^

While often more helpful than imaging in primary investigation of GI complaints, OGD is unable to rule out a diagnosis of Morgagni hernia. This is true even when stomach has herniated through the defect and particularly so when the hernial sac contains omentum or other abdominal viscera. In our patient repeated endoscopies over a year produced a range of outcomes, from essentially normal to suggested gastric volvulus.

Plain radiography may show air fluid levels in the herniated loops of bowel or stomach (lateral projection is particularly favourable for diagnosis); however the radiographic features are variable and depend on the contents of the hernia—omental contents will produce more subtle finding such as increased density at the cardiophrenic angles which can be misinterpreted as an epicardial fat pad. In prior case reports the frontal chest radiograph is often non-specifically abnormal, such as demonstrating a unilateral pleural effusion. Contrast fluoroscopy may also be normal.^1,7,10,11^ Though the diagnosis of Morgagni hernia is most commonly made incidentally on plain radiography, in the acute setting the findings are insufficiently sensitive or specific to be relied on in isolation. Thus where there is diagnostic uncertainty or a clinical suspicion of diaphragmatic hernia, CT is the gold standard for investigation due to the near-perfect sensitivity and allowing for detailed surgical planning.^1,10^

Once discovered, even if incidentally, it is recommended that Morgagni hernias are fixed due to the risk of future complications and the increased difficulty and poorer outcomes associated with acute repair. Laparoscopic mesh repair has become favoured in recent times with low complication rates and reduced hospital stays over the traditional laparotomy or thoracotomy approaches.^9^

## Conclusions

Despite being a relatively rare entity and classified as “congenital”, there are a growing number of case reports of elderly patients symptomatically presenting with anterior diaphragmatic (Morgagni) hernias. In many patients, as with this lady, the diagnosis is delayed as it is rarely considered as a potential cause and this can lead to poorer outcomes. Plain radiography may detect the diagnosis incidentally, however findings are dependent on the hernia contents and often equivocal in the acute setting. CT provides definitive diagnosis and allows for surgical planning.

This lady was unusual in that she had bilateral Morgagni hernias which were asymptomatic until her 9th decade, prior to which she had not undergone any thoracic or abdominal imaging. Indeed, even during her first admission she had no imaging performed (instead an OGD was done which appeared to have found the cause of the bleeding—only on later OGDs was the complex anatomy appreciated and imaging sought).

It is important to be aware that these “congenital” hernias can present later in life. As life expectancy and risk factors such as obesity increase we are likely to see a commensurate rise in the number of diaphragmatic hernias presenting in adulthood. Where there is clinical suspicion of diaphragmatic hernia imaging with CT should be first line. Plain radiograph is less definitive and cannot be solely relied upon in the acute setting. While often first line for GI complaints, endoscopy is unable to rule out this diagnosis.

## Learning points

1. Morgagni hernias are an uncommon form of congenital hernia which typically present in adulthood and are an important, rare cause of bowel obstruction and strangulation.
2. Endoscopy cannot exclude the diagnosis. In the acute setting plain radiography may be abnormal but non-specific. CT is the gold standard.
3. There are increasing case reports in the literature and with increased life expectancy and obesity they are likely to become more prevalent.
4. Diagnosis is often delayed, particularly in older patients, with associated detrimental impact on outcomes.

## Consent

Written informed consent was obtained from the patient for publication of this case report, including accompanying images.
